# Cultural Differences in Acceptance, Accessibility and Applicability of a Shared Decision‐Making Intervention for Older Adults With Multiple Long‐Term Conditions: A Feasibility Study Using Think Aloud Interviews

**DOI:** 10.1111/hex.70969

**Published:** 2026-11-13

**Authors:** Andrew K. Palmer, Suzanne Richards, Umesh T. Kadam, Karen L. Mattick, Florence Rogers, Lynn Tatnell, Beccy Summers, Joanne E. Butterworth

**Affiliations:** ^1^ Exeter Collaboration for Academic Primary Care (APEx), Department of Health and Community Sciences University of Exeter, St Lukes Campus Exeter UK; ^2^ European Centre for Environment and Human Health, University of Exeter Penryn UK; ^3^ Faculty of Medicine and Health, University of Leeds Leeds UK; ^4^ Department of Health and Community Sciences University of Exeter, St Lukes Campus Exeter Exeter UK; ^5^ Independent Public Collaborator, University of Exeter Exeter UK; ^6^ Patient and Public Involvement and Engagement Team, Applied Research Collaboration South West (ARC SW), University of Exeter Exeter UK

**Keywords:** cultural competence, health inequalities, multimorbidity, patient and public involvement, patient‐centred care, qualitative research, shared decision‐making

## Abstract

**Background:**

Shared decision‐making between patient and clinicians is crucial for older adults with multiple long‐term conditions, particularly those from culturally diverse, under‐represented groups who face health inequalities.

**Objective:**

This study evaluates the inter‐cultural feasibility of patient‐facing materials from a complex shared decision‐making intervention among culturally diverse older adults living with multiple long‐term health conditions.

**Method:**

Qualitative think aloud interviews were conducted with 20 participants aged 65 and older from White British, Afro‐Caribbean, and South Asian heritages across urban, rural, and coastal settings. We used thematic analysis to explore feasibility issues and inform the development of targeted approaches in the developing of intervention materials.

**Results:**

Our results demonstrate a range of diverse perspectives with respect to our intervention materials, from how the presence of inclusive messaging may mediate initial acceptance to how pressures on the health care system can affect relational continuity, which in turn undermines the efficacy of this messaging. Our data also demonstrates the importance of point‐of‐care delivery and the potential risks associated with transitioning from analogue to digital. The role family and third parties in care coordination was also observed as important factors in the design of interventions and the process of shared decision‐making more broadly.

**Conclusions:**

Our study reinforces the importance of seeking diverse perspectives to improve patient‐facing materials to ensure alignment between patients' lived realities and the healthcare provider's messaging. Reflecting the complexity of shared decision‐making as an intervention, the suitability of our patient‐facing materials centres around its visual acceptability, structural applicability, and intersectional accessibility. Targeted approaches are needed to involve diverse patients and carers in shared decision‐making, particularly given current changes to UK healthcare policy, and the need to address health and care inequalities among older adults with multiple long‐term conditions.

**Lived Experience Contribution:**

A Patient and Public Involvement (PPI) group, including twelve individuals with multiple long‐term conditions and/or carers, influenced all study stages from refining the research questions to interpreting results. A public representative and a PPI lead are listed as co‐authors on this manuscript and our public‐facing infographic, created with the help of these members, is available here: https://view.genially.com/6a9049b186120ca2ef1731db.

## 1 Introduction

The importance of shared decision‐making (SDM)—to actively involve patients in discussions about care and weigh up the clinical options and evidence alongside an individual's values and preferences—is increasingly recognised [1]. By embracing this shift from historical, paternalistic models toward relational, patient‐centred care, healthcare providers can enhance trust, satisfaction, and treatment adherence, while simultaneously uncovering unmet health and care needs [2]. People are living longer but spending more of their life in poor health due to the rising prevalence of multiple long‐term conditions (MLTC), which subsequently demands more intricate, person‐centred health and social support systems [3]. However, despite endorsement of SDM (as an ethically and morally grounded approach to consultation) [4], translating recommendations into routine clinical practice remains challenging.

Decision‐making is particularly complex for older adults living with MLTC, who commonly face cognitive or physical impairments, and competing treatment needs that are spread across multiple providers and carers [5, 6]. These older adults may need support from health and social care professionals who may invite participation and elicit their values, priorities and goals [5, 7, 8]. This support enables patients to voice their individual preferences and consider their priorities, relating to healthcare decision‐making, holistically [9]. However, although patient involvement is increasingly emphasised in guidance for managing MLTC, few interventions provide context‐specific and practical support for facilitating SDM with older patients [5, 10, 11]. This gap is further compounded by shifting UK demographics over the past decade, including the increasing cultural diversity of older age groups [10] and stark socioeconomic health inequalities [12]. Therefore, as society diversifies, a one‐size‐fits‐all approach may risk exacerbating inequalities by ignoring the cultural, economic, and health literacy factors that form how patients engage with care.

Healthcare access and shared decision‐making are shaped by a complex interplay of demographic, spatial, and technological barriers. Ethnically minoritised populations experience marked health inequalities which are underpinned by socioeconomic disadvantages [12] and earlier onset of MLTC for several ethnic minority groups [13, 14]. Obstacles to communication, such as language differences, low health literacy, and a lack of cultural competence in healthcare may in turn also further hinder engagement. For rural communities and older populations, these challenges may also be deepened by physical separation, as more centralised services and limited public transport constrain access to consultations [15]. Moreover, with the increasing prevalence of online systems and digital communication, those with a lack of devices or digital literacy are often excluded [16]. Together, these compounding factors leave underserved populations at a distinct disadvantage when navigating complex care decisions.

One approach to supporting these groups involves targeted interventions that modify a communication or implementation strategy to match the specific needs or unique context of a group or local setting. This moves away from a standardised procedure toward a dynamic, context‐dependent process. Designing these interventions often relies on audience segmentation that involves grouping potential users by meaningful demographic, cultural, or psychological characteristics to ensure information is culturally resonant and stems from trusted channels [17]. While tailoring can help the message land more effectively, in SDM interventions, the purpose is to open pathways to dialogue and collaboration rather than simply delivering information. This is especially relevant when SDM interventions are conceptualised as complex socioecological interventions that extend beyond a one‐off consultation [11]. However, opening these pathways also requires overcoming barriers such as language and health literacy. If patient‐facing materials rely on dense text or confusing layouts, they can impose an excessive cognitive load (i.e., the mental effort required just to process information [18]). Yet even with well‐designed, accessible materials, effective support for minoritised and marginalised populations in SDM must also be tailored to an individual's values, preferences, and support mechanisms. Without this, materials risk being rejected as culturally dissonant, and risk causing unintended harm by eroding trust and disincentivising future engagement [19].

This study evaluates the inter‐cultural acceptance of patient‐facing materials developed for the VOLITION project [8], which is a complex, multi‐level SDM intervention designed to support older adults living with multiple long‐term conditions by targeting both patients and practitioners. Using think aloud interviews [20, 21], with participants from diverse cultural backgrounds, we focus on how patient‐facing materials are both received and situated into the broader context of SDM. In doing so, we offer recommendations for targeted approaches within complex person‐centred interventions to meet the needs of diverse populations.

## 2 Materials and Methods

### 2.1 Study Design

This study was conducted within the pre‐implementation co‐production and refinement phase of the VOLITION project [8]. Following a Person‐Based Approach [20], we conducted think aloud interviews^20,21^ in which participants said out loud any thoughts that came to mind as they worked through it. This design was chosen to enable us to make suggestions that inform localised and target approaches with under‐represented groups and ensure the content can be translated into an acceptable, usable, and culturally sensitive tool. We do not aim to tailor separate versions for each group, nor a one‐size‐fits‐all intervention; rather, we explore how well our core version works across groups, and where targeting is needed for different settings and delivery formats. We adopted the COREQ [22]. checklist in writing this manuscript.

### 2.2 Patient and Public Involvement

Patient and public involvement was embedded throughout this study, aligning with UK Standards for Public Involvement [23]. The twelve members of our PPI group represented a broad range of cultural backgrounds from various geographic areas, with experience of living with or caring for those with MLTC. The group were active partners in shaping the study design, intervention materials, recruitment strategy, and analysis, and engaged through online workshops and email exchanges. The PPI contributions were tracked using an adapted version of PIRIT [24] and are reported in Supplementary File S1.

### 2.3 Intervention Materials

The patient‐facing materials component of the intervention consists of a four‐page physical leaflet, currently, in‐development, designed to (i) invite patients to take their preferred role in decision‐making and (ii) convey their personal priorities surrounding a decision to be made, particularly during consultations with their GP (see Figure 1). The development of the leaflet was guided by Intervention Mapping [8] and integrated patient, public, and stakeholder perspectives alongside evidence and theory [8]. The leaflet could be made available in GP clinics or disseminated within the community, for example through community organisations. The leaflet includes six example phrases designed to help patients express their personal preferences for involvement along a spectrum of collaborative styles. Using the patient‐facing materials alongside the provider‐training component of the VOLITION intervention, targets behaviour change in the receiver of the leaflet (addressing the determinants of patient knowledge, attitudes, perceived norms, self‐efficacy, and outcome expectations) [8].

**Figure 1:**
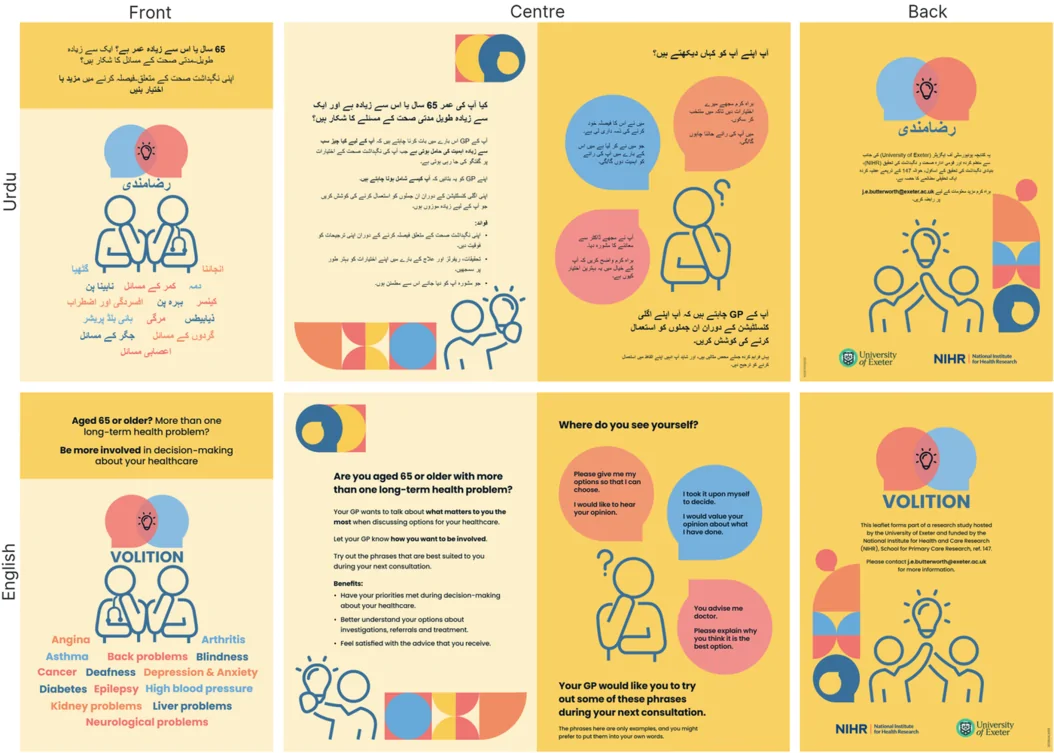
Patient‐facing materials in Urdu and English.

### 2.4 Sampling and Recruitment

Twenty participants living with MLTC were recruited through collaborations with the Race Equality Network, West Yorkshire, and the Research Engagement Network within the Health Innovation South West network, whereby potential participants were invited by known gatekeepers within these organisations and networks. The sample size was deemed sufficient to explore where responses to the leaflet diverge, rather than to compare perspectives between groups, which is in line with the concept of information power [25] and similar instructive studies [26, 27, 28]. We recruited individuals from South Asian, Afro‐Caribbean and White British ethnicities, from both urban and rural locations, including low‐income areas, as illustrated in Table 1. Broadly, our research captures the responses of ethnic minority participants from two communities, alongside perspectives from rural and coastal areas. It was not possible to approach people representative of all minority groups. However, all of the groups from which participants were recruited are known to experience health inequalities [12, 34]. Our design was intended to provide rich, new insights into these contexts with potential to transfer learning to other populations experiencing similar inequalities.

**Table 1:** Participant characteristics.

Research settings	Ethnicity*	Gender		Age Range	Interview support notes**	Mode
		Female	Male			
West Yorkshire A multi‐ethnic, urban community, featuring low‐income areas, and pressure on GP appointments through waiting times and staff shortages [29, 30, 31].	South Asian (*n* = 6)	2	4	65–88	1 facilitator for all participants. 2 carers 2 family members	MS Teams and WhatsApp video calls
	Afro‐Caribbean (*n* = 5)	3	2	69–89	1 facilitator for all participants	MS Teams
Devon & Cornwall Rural and coastal areas that feature low population density, low‐wage economy and geographic isolation [32, 33].	White British (8)/Cornish (1) (n = 9)	6	3	65–86	1 carer/family member	In‐person and telephone

* Participants self‐ascribed their ethnicity.

** Facilitators from our collaborators provided technical and personal support.

All participants were encouraged to be supported during the interview by carers, and those taking part as a participant or in a supportive role provided informed written consent, either physically or online. Participants and carers/facilitators were offered the NIHR standard rate reimbursement to thank them for their time [35]. Ethical approval was granted by The University of Exeter Medical School's Faculty of Health and Life Sciences Ethics Committee (ID: 11249357).

### 2.5 Think Aloud Interviews

Think aloud interviews [20] were conducted by the first author, a White male research associate, with non‐clinical research experience in health inequalities and under‐representation. He was introduced to the participants in West Yorkshire by our collaborators and met participants in the Southwest by attending pre‐arranged community group events. The interviews took place either via video conferencing, in‐person, or over the phone. The development of the interview guide was informed by the Person‐Based Approach [20] to study users' real‐time experiences. It consisted of two sections—the participant‐led think aloud section—and a follow‐up semi‐structured interview section [20]. During the think aloud section, participants were asked to talk through their thoughts as they interacted with the patient‐facing materials. The South Asian participants were provided an Urdu version, due to it being the prevalent language in this community. All other participants received the English version. The open‐ended section consisted of six questions to explore aspects of the intervention's feasibility [20, 36]. Participants were provided with the leaflet prior to the interview through our collaborators or in‐person to reduce uncertainty and to enable more considered responses [20].

### 2.6 Analysis

Interviews were audio‐recorded and transcribed professionally, and then analysed in NVivo 15 following Braun and Clarke's [37, 38] guidelines for reflexive thematic analysis. The first author coded the transcripts for meaningful or significant insights with respect to the project aims. Several hundred initial codes were generated and sorted into higher‐order interpretive categories. A second analyst (a female academic clinical fellow with experience in inductive thematic analysis) was provided with this category framework and coded three transcripts. She was encouraged to look for nuance, differences and disconfirming cases, and to generate new codes where the framework did not fit. This process was used as a collaborative tool to broaden interpretation, challenge assumptions and refine the themes. The categories were presented and discussed with the PPI members to aid and broaden the interpretation such as helping us sharpen and distil our themes and the key messages.

## 3 Results and Discussion

Our findings are structured around seven interconnected themes derived from our thematic analysis (Figure 2). In what follows, we discuss these themes in the context of relevant literature before concluding with a reflection on how they collectively inform implications for practice and the implementation of complex person‐centred interventions.

**Figure 2:**
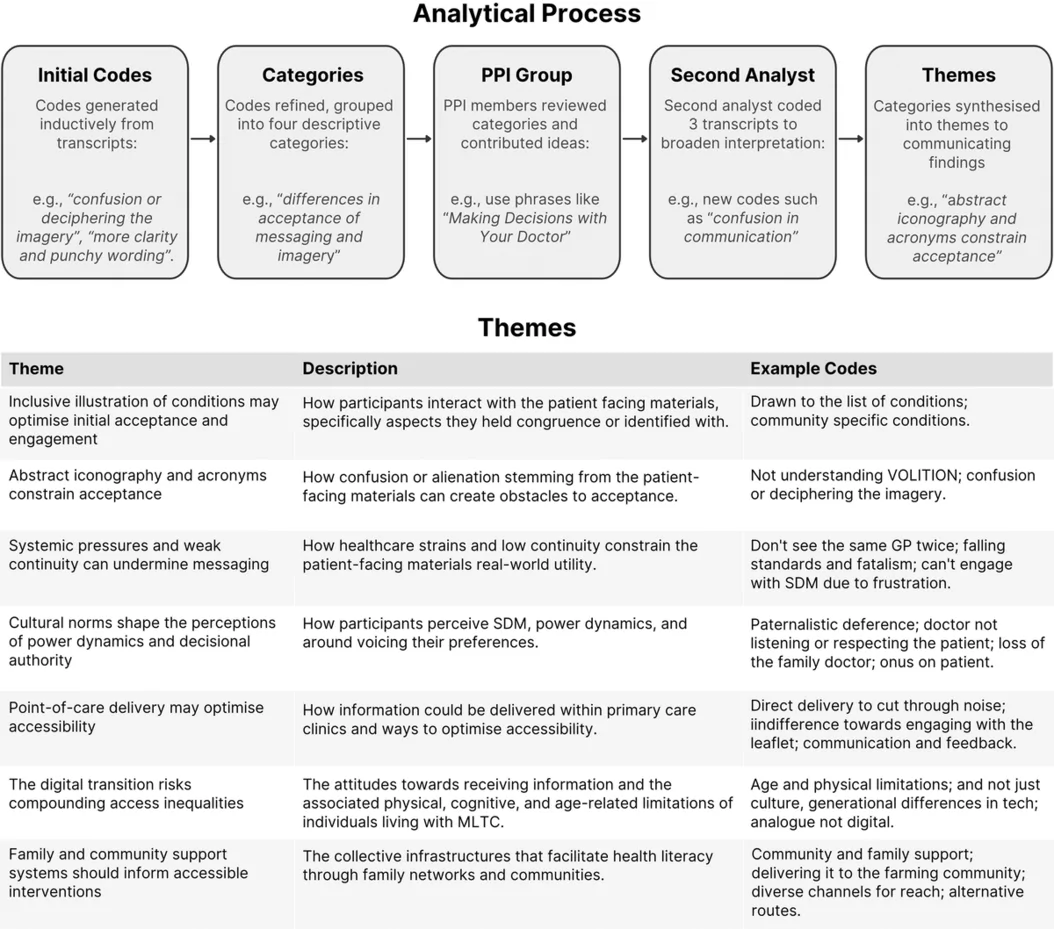
Illustration of analytical process and themes.

### 3.1 Inclusive Illustration of Conditions May Optimise Initial Acceptance and Engagement

The interviews were useful in revealing the real‐time responses of participants as they interacted with the leaflet. We found that for all ethnic and geographic groups, the word‐collage depicting various long‐term conditions was effective at creating this point of resonance and personal identification:

I can see that, me diabetes, that relates to me.

(B10, Caribbean, M, 65‐69)

…they just went straight on to the words that, the next bit, the ailments […] I think it's because they look at it, they see the words, they can relate to it.

(B7, Caribbean, F, 85‐89 ‐ Facilitator)

…arthritis and back problems, they're both very much involved in the farming […] depression is another big thing with the farming community.

(D1, White British, F, 85‐89)

I think it's good for Asian people. Basically, this leaflet is giving awareness […] we can talk about issues [where] sometimes we don't talk, like, especially […] about depression, we don't talk about this.

(B5, South Asian, F, 70‐74 ‐ Facilitator)

As seen above, diagnostic terms functioned as visual heuristics, allowing participants to quickly ascertain if the material was relevant and providing a cognitive shortcut to acceptance. Whilst this uncovers useful applications for leveraging illustrations of conditions, this approach must be balanced to avoid over‐targeting which risks reproducing health inequalities by inadvertently excluding individuals whose profiles fall outside the chosen categories [39, 40]. This ‘double‐edged sword' was also identified by PPI members who stated that whilst particular conditions could draw individuals in, it risked alienating patients whose specific conditions are omitted. To mitigate, the PPI panel suggested including overarching terms such as “multiple long‐term conditions” to increase the inclusivity.

### 3.2 Abstract Iconography and Acronyms Constrain Acceptance

Whilst there was broad acceptance of the leaflet's overall design and purpose, variations emerged in how well the participants responded to specific elements. Some of the language and iconography did not resonate with several participants from minoritised ethnic backgrounds, who experienced difficulty decoding some of the symbols or navigating the phrasing. The quotes below detail this confusion, including B8's assessment of whether the central illustration of the doctor and patient shown in Figure 1 was a suitcase, which in itself could carry unintended negative meanings:

Oh, the colour's good, ‘cos it's an attractive colour, but that's all. It doesn't, do you know what I mean, it's attractive but the words or whatever it's using, if it was something more ‐ what‐have‐you ‐ easy to understand — it would be better.

(B10, Caribbean, M, 65‐69)

I wouldn't figure that out by myself. Apart from the question mark, I would know that. But like a bulb, you know what I mean, those sort of things I wouldn't know.

(B11, Caribbean, F, 85‐89)

I noticed that [image] and I'm wondering because is the person pushing a suitcase? [laughs].

(B8, Caribbean, M, 80‐84)

The quotes above demonstrate the importance of testing this intervention, especially when considering the high proportion of individuals facing health inequalities who speak English as an additional language [41, 42]. Moreover, icons are often culturally bound symbols that require specific knowledge to decode [43] and western‐style imagery, such as a lightbulb used to signify an ‘idea', may inadvertently exclude the groups the intervention seeks to engage. This issue was also critiqued by our PPI contributors, who added that imagery can carry unintended biases such as how an image of a clinician being larger than a patient could signal a power dynamic that undermines the collaborative message. Both participants and the PPI group recommended replacing overly polished designs with simpler elements, like cartoon‐like speech bubbles, to improve acceptability. This echoes useful research that demonstrates how complex layouts have been found to reduce an intervention's effectiveness for lower‐literacy groups, whereas concise, plain‐language designs perform better [44].

We also found that the placement of the project name, “Volition”, alienated several participants, who found the term to be confusing, and therefore produced an effect diametrically opposed to that of the word‐collage:

That don't tell me jack! I just don't understand what that word is! [Laughs] What's the point of opening summat or trying to think of something if I don't know what the word means.

(B10, Caribbean, M, 65‐69)

‘Volition', I have no idea what that means or why it's called that or what it's trying to tell me […] To put that as your biggest headline in the middle of the page, um, will immediately alienate people.

(C4, White British, M, 65‐69)

The frustration with a term that is uncommonly used in British vernacular prompts us to reflect on how the use of acronyms, which is oft‐encouraged by funding bodies, can seep into materials with unwanted effects [45, 46]. Our PPI panel responded with advice about how future iterations could adopt action‐oriented headers such as “*Making Decisions with Your Doctor*” to avoid these constraints.

### 3.3 Systemic Pressures Affecting Continuity Can Undermine Messaging

We found that participants' perceived applicability of the leaflet depended heavily on their socio‐cultural and structural realities [11]. Participants frequently situated their experiences within a broader climate of perceived falling standards in the NHS. Interviews were characterised by recurrent complaints about long waiting times—for example, one participant noted, “I get fed up of waiting on the phone, wasting my time, so I don't ring doctors anymore” (B8, Caribbean, M, 80‐84). Others highlighted the receptionist's role as an obstructive gatekeeper, with one asking: “She's not a doctor, so why should you have to go through all that before I get to see a doctor?” (B10, Caribbean, M, 65‐69). In line with existing literature, this phenomenon reflects the perceived discretionary rationing of care by staff [47]. Our data demonstrates that these barriers compromised the efficacy of the leaflet itself by rendering it a seemingly futile gesture:

I mean it's very nice putting the leaflet and everything, and I do not dispute that but if, for example, we are not following that what they are saying on the leaflet, the leaflet is doing nothing. The leaflet is doing nothing, if the patients are not getting this outcome, the leaflet is very nice yes, because we have got to get the information, but when you ask the question, there is no one to answer that question. You talk to you receptionist “oh you need to talk to your doctor about that”, but you don't get to see the doctor to ask that.

(B1, South Asian, M, 70‐74)

While the insight detailed by B1 informs both our methodological and conceptual thinking, it moves us to consider how the practical application of such interventions requires deeper reflection. Whilst it has been noted how the fidelity of an intervention does not inherently guarantee patient engagement [48], there is a need for congruence between the lived realities of patients and the messaging that extends from practitioners through the intervention. Nuances in our data reveal highly differential experiences, with participants alluding to geographic variations, perceptions of lower standards of care and staff withholding appointments.

Yeah, he is saying it's tricky, it's tricky. Yeh because you know [in our city], the situation is different, because people are angry because doctors are not giving us any appointment, and they are all getting frustrated, so it's a bit of a concern.

(B1, South Asian, M, 65‐69 ‐ Facilitator)

I'm going there and they were just running around, and I was thinking why we have less staff compared to the other England parts.

(B5, South Asian, F, 70‐74)

Consequently, inter‐cultural feasibility demands authentic, transparent communication by signalling a genuine intent to engage and reassuring patients that clinicians are seeking to provide care within these constraints. C4 (White British, M, 65‐69) noted how patients disengage if text describes “an experience they've never had”. He suggested minor semantic adjustments like rephrasing to “A GP” or “Our GPs” to better match his experience of primary care—an observation that is supported by broader health services research demonstrating how a decline in continuity is linked to an increase in patient frustration and complaints [49]. In response, approaches that harness those realistic, everyday encounters, may be fruitful in this domain. Looking forward, policy initiatives such as the NHS 10‐Year Health Plan for England [50] may reshape this context with plans to transition toward better coordinated, community‐based care that increases relational continuity.

### 3.4 Cultural Norms Shape the Perceptions of Power Dynamics and Decisional Authority

Participants frequently lamented the ‘loss of the family doctor,' a historical model of care in which trust and intergenerational family knowledge were more abundant. Some of the Caribbean participants reflected on these shifts, noting how years of systemic restructuring have eroded these connections, leaving them to navigate superficial, more transactional relationships:

You've got to be able to open up to that doctor and you can't because your doctor is not your doctor from when you were a child. […] Your doctor would already know who you are ‘cos he's your family doctor […]. Like now, he wouldn't even know who the bloody hell I am!

(B10, Caribbean, M, 65‐69)

This sense of anonymity is further compounded by a pervasive narrative of time scarcity within the current primary care landscape, which contrasts with accounts of the unhurried, relational spaces of the past:

In the early days, the sixties, the seventies, the eighties, your doctor used to call you in and then you and him would have a chat. […] But these days, as soon as you walk in, you're out. […] Everybody's rushing, you know, so before you would come in the surgery […] sit down and have a little talk, you know. You don't get this anymore.

(B8, Caribbean, M, 80‐84)

This disconnection encompassed discussions of the leaflet's design. Many participants spoke of superficial relationships with doctors, not being listened to, or even facing disrespect, meaning the use of the leaflet's toolkit (example phrases) might need more specific targeting for these communities. For example, there was a feeling amongst most participants that whilst they were open to SDM, as evidenced by the positive sentiment toward the benefits, the doctor had not explicitly elicited these values or invited them. This was combined with the fact that the leaflet, to them, seemingly put the onus on the patient for initiating SDM, which was said could be altered to be more direct, relational and congruent:

Um, I still feel at arm's length with my GP, um, I mean, in terms of decision making.

(C1, White British, F, 80‐84)

[*on whether the phrase “Are you listening to me?” on the leaflet would be attractive*] That would attract me! Because that's what I'd want to know, is why you're not listening to me!

(B10, Caribbean, M, 65‐69)

This preference for a direct, emotive hook highlights how outreach interacts with cultural perceptions of SDM. When evaluating the spectrum of preferences for involvement, nearly all participants sought a middle ground where they could receive both choices and guidance. Crucially, participants often perceived the more assertive phrases on the leaflet as taking a “rude” or “brave” stance against the practitioner. The participants' interpretation of the text highlights a need to refine the messaging. It suggests that while the intervention aims to identify how a patient prefers to navigate a consultation, the language must be adapted so it is not misconstrued as an attempt to ignore the doctor's experience.

To ensure these materials are applicable, closer attention must be paid to how patients perceive and navigate clinical power dynamics. The concept of “power distance” [51], which is the extent to which a society expects and accepts unequal power distribution, is a useful interpretive lens here. A low power‐distance orientation aligns with a meeting of equals, whereas high power‐distance positions the clinician as an authority. While some participants' deference may point to a desire not to overburden a busy clinician, existing literature suggests that for minoritised groups, similar behaviours are multi‐faceted. For example, power‐distance orientation may be rooted in professional respect [52], or alternatively, a protective distance stemming from historical and systemic distrust of healthcare institutions [53]. While our findings do not allow us to attribute specific motivations to individual demographic groups, they highlight the potential of considering these nuanced power dynamics in future communication strategies.

### 3.5 Point‐Of‐Care Delivery May Optimise Accessibility

The interviews revealed insights into how the leaflet was received in real time, with participants relaying first impressions and reflecting on whether the leaflet resonated with them. The leaflet's accessibility was set against a backdrop of busy surgeries with large volumes of patient health information on noticeboards and leaflet racks. Pertinently, a solution was proposed by some participants was more direct modes that utilised the leaflet's efficacy as a SDM tool:

Sometimes the noticeboard in the doctor's surgery is usually jam‐packed with leaflets for things. You know, notices rather than leaflets, to look at.

(D1, White British, F, 85‐89)

I think, actually, if you're going for a consultation, to give it you while you're sitting waiting […] I think that's a good idea ‘cos it's focusing you.

(A1, White British, F, 80‐84)

Yeah, I mean, if they're left in the reception area when you're waiting, then I never pick anything up from there, really… If the receptionist gave me that, then I would obviously look at it and read it, yeah. (C2, Cornish, F, 70‐74)…it's probably not the first thing you'd pick up, it'd have to be really customised for him, meaning somebody handed it to him, rather than him fishing it out.

(B6, South Asian, M, 70‐74 ‐ Carer)

Whilst not necessarily practical in all clinical contexts, having the leaflet handed to patients at the point‐of‐care before a consultation, or provided in‐person during visits, would enable them to digest the content and adopt the illustrative phrases underpinning the intervention. This approach may be particularly vital for addressing entrenched health inequalities and counteracting clinical stereotyping. While younger patients are often assumed to be natural information seekers who desire autonomous decision‐making, older adults living with MLTC frequently rely on a trusted practitioner ally to help navigate and funnel increasingly complex health information within the context of their daily lives [1, 2].

### 3.6 The Digital Transition Risks Compounding Access Inequalities

Our findings underscore that a transition toward exclusively digital healthcare communication risks exacerbating existing inequalities, which counters the UK policy agenda's focus on inclusive transformation. Instead, participants in this study demonstrated a wide spectrum of preferences, as illustrated by their varying responses to different formats:

This format thing where you can, digital, on your phone, that's such a nightmare […] not all old people of my age of sixty‐nine, are used to formats. But reading something like that rather than going on the internet or the thing on the computer and try to get it up there. Not all of us is good enough to do that.

(B10, Caribbean, M, 65‐69)

He would prefer paper, he doesn't do emails, he doesn't do texts, he doesn't even answer his phone!

(B8, Caribbean, M, 80‐84 ‐ Facilitator)

Text is best option because at least you know you see the thing in front of you, and you can analyse it and things like that. Most of the time as it is a leaflet, it is human nature you pick it up and then you put it on the side, with the text is there you just look at it.

(B3, South Asian, M, 65‐69)

OK, she said that she would like a video as it would help her visually understand, ‘cos she'd be able to rewatch the video and the leaflet as well.

(B2, South Asian, F, 65‐69 ‐ Carer)

To translate these insights into actionable inter‐cultural intervention strategies, health providers can benefit from employing intersectional frameworks, i.e., recognition of different and mutually constitutive aspects of one's identity [54, 55]. Our findings strongly suggest that the very acceptance and usability of patient‐facing materials are driven by intersectionality. As participant B8 exemplifies, digital pathways can exclude certain vulnerable populations. These digital barriers then intersect with diverse cultural practices, demographic transitions, and the cognitive or physical limitations inherent in MLTC. The case for tailored, multi‐channel delivery becomes even more of a necessity, which is evidenced here and elsewhere in our data in how these distinct axes of age, ethnicity, physical illness, and geography collide to determine whether a patient can engage with the material or if it becomes inaccessible to them:

Lots of people like it online, but I think as you get older and your eyesight's deteriorating, reading a screen is quite difficult, really. So, I do think—and also when you're getting older, you forget more, do you know what I mean, it takes you longer to understand things.

(A1, White British, F, 80‐84)

His eyesight is also [bad]… so the leaflet in this way would be very low down the scale… Visual aid could help, but as a secondary means… primary would be verbal [through me].

(B6, South Asian, M, 70‐74 ‐ Carer)

As we discussed in the opening section of this paper, we are experiencing a rapid shift towards digitisation driven by society and government. In England, this tension is brought into focus by the government's NHS 10‐Year Health Plan [50], which explicitly mandates a structural shift “from analogue to digital” across the health service. This means that practitioners must be sensitive to diverse needs and responsive to a changing context. This includes more focused attention on how to make digital or multi‐channel tools feasible ‐ findings of a recent qualitative systematic review [56] show that for such tools to succeed amongst physical limitations and issues of digital literacy, primary care teams must seek to provide initial training, tailor the tools to the patient's lived experience, and facilitate a supportive dialogue.

### 3.7 Family and Community Support Systems Should Inform Accessible Interventions

The intervention's feasibility was rooted in individual and community contexts, highlighting key cultural differences that practitioners should recognise. Not only was the role of carer evident, as seen above (B6's carer), but family support systems such as children and partners were also important mediators in health information and care more generally. This emerged with prominence in discussion with members of the Caribbean community, who described their children supporting them in an increasingly complex world:

Well now, I don't talk to the GP, I don't talk to the heart nurse, it's all my daughter, everything is from the heart nurse, the consultant and my daughter, anything that has to be changed, medication, I pass it on to me daughter, my daughter passes it on to me. And I'm a happy baby.

(B7, Caribbean, F, 85‐89)

In general, what we do, especially with the Afro‐Caribbean community, when our parents get old, we then take the children […] if you see GPs you always find if there's a senior, there's somebody with them […] the paper's fine for the seniors, but then there should be something, like you say, an email, like today, if you want to send her an email, the email will go to her daughter.

(B7, Caribbean, F, 85‐89 ‐ Facilitator)

The insights provided by B7 and her facilitator during the interview demonstrate that involving members of this cohort in SDM may also include engaging family members and making them welcome. It was even remarked that specific messaging could be used, such as “Are your parents suffering from this?” (B7, Facilitator). This push towards intergenerational messaging moves the intervention to a triadic framework of care. In a scoping review of SDM among minoritised populations [57, 58], it was found in one study that “family members played a central role in decision making, with patients sometimes deferring to their spouses or children for the final decision” (p.112). Taking this into consideration, the preceding messaging may also require this kind of messaging, which extends to both formal and informal carers—the PPI members added complementary insights stating that carers should be acknowledged and included in messaging. These tailored approaches ensure the intervention moves from an individual burden to a community asset, and that cultural applicability and feasibility is built into wider support systems.

Whilst we have seen that much of the data hold tension with a rapidly changing UK context, we also found alignment in how these modes and support systems meet current national and international policy priorities. Rather than relying solely on traditional primary care models, our findings support broader health policy aims to shift care out of institutional settings and into the community [59]. Highlighting the limitations of relying on surgeries for rural groups, D1 (White British, F, 85‐89) noted: “I don't know that farmers would particularly notice it at a doctors' surgery.I know my husband wouldn't have bothered to look”. This disconnect was echoed across the cohort by participants who rarely visit the surgery. C4 (White British, M, 65‐69) mentioned that “most of the things that I take any interest in, are not in the surgery, because I don't often visit the surgery,” and C3 (White British, F, 65‐69), who remarked, “I haven't walked into my doctor's surgery since last July, so I would have no knowledge of this actually being available.” Instead, shifting the intervention to more active communal spaces where people naturally gather may offer a higher chance of engagement:

the market area […] or perhaps the café area at the market [works because] the market is a good place for farmers to notice things.

(D1, White British, F, 85‐89)

A bigger format for, you know, like community centres or the GPs practices or maybe the schools and give a lot of information about…to the communities.

(B3, South Asian, M, 65‐69)

I think having this out in the open, in different, I don't know, things like the pain clinic yesterday or community centres […] there's the council buildings, there's a pain café, there's community centres and even a lot of villages have community centres or even in churches.

(C3, White British, F, 65‐69)

The data above speaks to a sociology of health information dissemination through third places ‐ the neutral, informal locations like markets, cafés, and faith groups that exist outside of home and work [60]. In a qualitative study on healthy ageing [61], it was found that informal public settings function as social infrastructure where casual, everyday encounters with familiar faces or strangers build a sense of local belonging and personal safety. This means that healthcare interventions can tap into these environments where older adults feel more receptive. However, caution is required because this research did not examine culturally diverse populations. Place‐based approaches may nonetheless broaden access to health initiatives beyond GP surgeries and make them more inclusive and culturally acceptable [62].

### 3.8 Strengths AND Limitations

The study captured responses from older adults with MLTC across a purposively sampled range of ethnic, socio‐economic and geographic backgrounds, which provided insight into the leaflet's acceptability and applicability, and into how patients engage with health information and broader healthcare structures. The sample was designed to identify issues with a single (patient‐facing) aspect of the VOLITION prototype intervention, and we judge it sufficiently information‐rich for this aim [25]. However, it was not designed to compare groups, and numbers are small when disaggregated by identity variables, which precludes granular, group‐specific recommendations. Therefore, findings should be read as identifying issues that may differ across groups. Relying on facilitators and caregivers for some non‐English‐speaking participants may also have constrained our understanding of cultural nuances, even though they were drawn from trusted community organisations. PPI throughout the study strengthened the relevance of our interpretation and recommendations, which helped to mitigate these limitations.

## 4 Conclusions AND Implications

The aim of this study was to generate empirical evidence on how diverse groups experience shared decision‐making patient‐facing materials. Against a UK healthcare backdrop of accelerating digitisation, shifting relational continuity, and persistent socio‐geographic inequalities, our central finding is the need for congruence between the intervention's messaging and the diverse, lived realities of patients and their carers'. These findings come from older adults living with MLTC, whose accounts were shaped by their later‐life circumstances, including reliance on family members to coordinate care, limited familiarity with digital formats, and for some, a sense of lost relational continuity. We now synthesise our conclusions around the tool's visual acceptance, its structural applicability, and its intersectional accessibility, which reinforce SDM's conceptualisation as a complex socioecological intervention [11].

The visual acceptance of the function of our patient‐facing materials is crucial, as they provide a physical aid to invite patients to be more involved in decision‐making about their care. This gateway depends on inclusive messaging that participants can identify with, which requires simpler visual and written elements. Most importantly, the co‐production of materials will aid local and culturally nuanced interventions that may avoid these obstacles. Moreover, we must avoid further reinforcing existing inequalities by adopting person‐centred, not condition‐centred, health communication that ensures that messaging does not worsen constraints for those with limited health literacy [6].

The structural applicability of the patient‐facing materials was revealed through insights in how participants are experiencing care. This demonstrates that structural pressures can pollute the real‐world applicability of patient‐facing materials. The findings dictate that we must be responsive and adopt more authentic messaging that matches the current constraints of primary care and recognises different cultural perceptions and power dynamics. This extends to ongoing work [8] that seeks to equip practitioners with the skills and knowledge to recognise and balance power dynamics to deliver stronger, culturally nuanced shared decision‐making. Operationalising this clinical side of the intervention is central to the NHS vision for care closer to home and for localised care coordination [63].

The need for intersectional accessibility was revealed through diverse findings reiterating the need for dynamic, system‐level, and interpersonal solutions that intersect with impending transitions for the NHS in England [64, 65]. For example, reaching people in remote locations or those who rarely visit primary care practices may be improved by embedding point‐of‐care delivery in community settings. The policy shift “from hospital to community” through Neighbourhood Health Teams provides a mechanism to deploy these localised and decentralised models [63, 64]. However, to ensure these models do not inadvertently widen health inequalities [66], they must be designed around local geography and travel constraints while instilling the trust necessary to engage in shared decision‐making [11].

To tailor this approach effectively, digital leaflets could be sent to patients or designated family members before their consultations, and complemented by, widely available physical materials. Adopting this into practice, may increase relational continuity while also working sensitively in line with the digital transition and avoiding digital‐by‐default policy that may lead to reluctant uptake [65, 67]. Healthcare providers must facilitate and enable this access to mitigate digital exclusion among vulnerable groups, which will require ongoing training to enable practitioners to conduct such outreach [63]. For older adults, this means welcoming family members and carers into the messaging and offering paper materials alongside digital ones.

## Author Contributions

**Andrew K Palmer:** investigation, writing – original draft, writing – review and editing, visualisation, methodology, formal analysis, data curation. **Suzanne Richards:** conceptualisation, methodology, funding acquisition, supervision. **Umesh T Kadam:** conceptualisation, methodology, funding acquisition, supervision, writing – review and editing. **Karen L Mattick:** conceptualisation, methodology, funding acquisition, supervision, writing – review and editing. **Florence Rogers:** formal analysis, writing – review and editing. **Lynn Tatnell:** conceptualisation, project administration, writing – review and editing, funding acquisition. **Beccy Summers:** conceptualisation, methodology, writing – review and editing, project administration, funding acquisition. **Joanne E Butterworth:** conceptualisation, methodology, funding acquisition, investigation, writing – review and editing, supervision.

## Ethics Statement

Approved by the University of Exeter Health and Life Sciences Research Ethics Committee (Ref: 11249357). All participants provided informed consent either in writing or online prior to participation.

## Conflicts of Interest

The authors declare no conflicts of interest.

## Supporting information


Supporting File

## Data Availability

Data are not publicly available due to the qualitative nature of the study and participant privacy requirements, as explicit consent for data sharing was not obtained.
