# Supplementary material for: Cultural Differences in Acceptance, Accessibility and Applicability of a Shared Decision‐Making Intervention for Older Adults With Multiple Long‐Term Conditions: A Feasibility Study Using Think Aloud Interviews
Source: Health Expect. 2026 Nov 13;29(6):e70969. doi: 10.1111/hex.70969 (PMC13768807; doi:10.1111/hex.70969)
Supplement: Supplementary file 1 [file HEX-29-e70969-s001.docx]

**VOLITION Patient and Public Involvement**

This short supplement provides additional detail on the involvement of public members on the NIHR-funded research project. Twelve public members, from a range of backgrounds and ages, were involved throughout the project. Public members contributed regularly into project decision making through a variety of formats, such as online group meetings, individual meetings and written input, in line with members preferences and requirements. The public co-applicant, and the PPI lead also attended monthly team meetings to discuss project progress, share PPI group input and inform overall project decision making. Table 1, below, provides examples of how public members impacted the project across all stages of the research cycle.

| Table 1 | |
| --- | --- |
| Pre-project setup and recruitment | - The public co-applicant was actively involved in project steering meetings, refined patient-facing documents, guided the vision for the intervention and reviewed the grant application for this project. - A public member and our PPI lead, acted as interview panel members when recruiting a postdoctoral researcher, who later led recruitment and data collection, and is subsequently the lead author of the manuscript. |
| Shaping the research questions | - The public co-applicant attended regular meetings with the research team to contribute to the design of the research questions. This built on previous shared decision-making work of which she was also a contributor. |
| Designing the methods | - Members helped revise the wording of multiple interview questions so that they were easier for people to understand and so that people would feel welcome and unhurried during the interview. - The group changed the way the researcher introduced themselves to participants at the start of the interview to better support building rapport with participants to create a safe and comfortable interview space. |
| Collecting the data | - The members were not involved in the collection of data due to practical constraints and the need to work with external organisations. Instead, we collaborated with Voluntary, Community, and Social Enterprise sector organisations (including the Race Equality Network, West Yorkshire, and the Research Engagement Network, South West) who helped recruit participants and facilitated data collection (think aloud interviews). |
| Analysing and interpreting the data | - Members informed the interpretation on why certain patient groups (particularly South Asian and Afro-Caribbean participants) struggled with abstract icons or speech-bubble graphics in the intervention leaflet, and what could be done to improve this aspect in the future. - They also discussed the strong role that family members/adult children play in managing appointments and health information for older relatives and help shape the recommendations in the main paper. |
| Sharing the findings | - Members evaluated a range of health infographics, posters, and leaflets to figure out what visual elements work best for them. Members recommended using clear, high-contrast colours and to avoid acronyms/jargon. They suggested using positive and encouraging language and using relatable imagery rather than more formal graphics. - Members informed the creation of an accessible interactive infographic that summarised the study findings and public involvement by providing feedback to designers at [Nifty Fox](https://www.niftyfoxcreative.com/) who presented a range of different design formats and options. The infographic is [available here](https://view.genially.com/6a9049b186120ca2ef1731db). - Members reviewed and co-authored the Plain Language Summary by providing feedback to the researchers and editing the text together. - The public co-applicant reviewed the submission abstract and materials for presenting this work at Society for Academic Primary Care conference 2026 in St Andrew’s. |

Public members were reimbursed through the standard NIHR participation payment rates for all involvement activities ([NIHR, 2025](https://www.nihr.ac.uk/about-us/who-we-are/policies-and-guidelines/payment-guidance-researchers-and-professionals)).
